# To evaluate the value of L1 cortical bone quantitative CT parameters in predicting osteoporosis

**DOI:** 10.3389/fmed.2026.1756961

**Published:** 2026-04-23

**Authors:** Qianqian Yao, Qiufeng Yao, Jian Qin, Fanghua Liu, Xin Chen, Hui Yang, Liqing Kang

**Affiliations:** 1 Graduate School, Tianjin Medical University, Tianjin, China; 2 Department of Radiology, The Second Affiliated Hospital of Shandong First Medical University, Taian, China; 3 Department of Pain Management, Jining Hospital of Integrated Traditional Chinese and Western Medicine, Jining, China; 4 Department of Magnetic Resonance Imaging, Cangzhou Central Hospital, Cangzhou Teaching Hospital of Tianjin Medical University, Cangzhou, China

**Keywords:** bone mineral density, cortical bone, osteoporosis, quantitative computed tomography, trabecular bone

## Abstract

**Objective:**

To explore the changing trends of cortical bone of the first lumbarvertebral body (L1_cortical bone_) measured by the CT spinal bone quantitative system with age, sex, and volume bone mineral density(vBMD), as well as their predictive value in osteoporosis.

**Methods:**

A retrospective analysis was conducted on 169 participants who underwent simultaneous chest-abdominal or lumbar spine computed tomography (CT) and quantitative computed tomography (QCT) scans. All participants were stratified by sex (male and female) and further divided into three age groups: 50–59 years, 60–69 years, and ≥ 70 years. Based on QCT results, participants were categorized into two groups: non-osteoporosis (including normal bone mass and osteopenia) and osteoporosis. The average density, average thickness, average area and the total volume of L1_cortical bone_ were measured using a CT spinal bone quantification system. A one-way analysis of variance (ANOVA) was employed to compare differences in the aforementioned parameters among different age and vBMD groups, while receiver operating characteristic (ROC) curve analysis was used to evaluate their efficacy in predicting osteoporosis.

**Results:**

For all participants and the female subgroup, there were statistically significant differences in the average thickness, average area, and total volume of L1_cortical bone_ among the 50–59, 60–69, and ≥ 70 year age groups, with all parameters showing a decreasing trend with increasing age. Additionally, statistically significant differences were observed in the average thickness, average area, and total volume of L1_cortical bone_ among the normal, osteopenia, and osteoporosis groups, and these parameters exhibited a decreasing trend with decreasing vBMD. In the male subgroup, the area under the ROC curve (AUC) values of the three parameters (average thickness, average area, and total volume) for detecting osteoporosis were 0.77, 0.83, and 0.84, respectively. The average thickness of L1_cortical bone_ demonstrated the highest sensitivity (82.89%), whereas the average area showed the highest specificity (90.00%). A similar trend was observed in the female participants.

**Conclusion:**

The quantitative parameters including the average thickness, average area, and total volume of L1_cortical bone_ measured by the CT spinal bone quantitative system appear to show a downward trend with increasing age and decreasing vBMD, and may potentially help in detecting osteoporosis.

## Introduction

Osteoporosis is a systemic skeletal disease primarily caused by an imbalance between osteoclast and osteoblast, which ultimately leads to pathological loss of bone mass (1). Clinically, it is mainly characterized by decreased bone mineral density (BMD), reduced bone mass, and is prone to complicated osteoporotic fractures (2). At present, approximately 32% of the population aged over 65 years in China suffer from osteoporosis, and it is estimated that the overall growth rate of osteoporosis population in China will reach 104.70% by 2050. Moreover, with the intensification of global population aging, osteoporosis has become a major global issue. Therefore, the early identification of osteoporosis and prevention of osteoporotic fractures will greatly improve the quality of life of patients, thereby further reducing the family economic pressure and social burden.

Previous studies on osteoporosis have mostly focused on trabecular bone (3–6). Recent studies have found that cortical bone also plays an important role in the occurrence and repair of osteoporosis. Bone biomechanical studies have shown that although most fractures in the vertebrae, femurs, and other parts occur within the trabecular bone, they initially originate from cortical bone defects (7). When trabecular bone mass is lost, the integrity of the connection between cortical bone and trabecular bone is disrupted, and cortical bone gradually becomes a key load-bearing component. Both the thinning of cortical bone thickness and the increase in cortical bone porosity play important roles in fractures. Furthermore, because the BMD measured in trabecular bone cannot detect the preferential orientation of collagen/apatite, trabecular bone can only predict 50–80% of vertebral fractures, a limitation that cortical bone can compensate for (8). Therefore, compared with trabecular bone, cortical bone has a closer association with bone strength and is more susceptible to mechanical forces (9).

Currently, the early screening and diagnosis of osteoporosis mainly rely on dual-energy X-ray absorptiometry (DXA) and quantitative computed tomography (QCT) (10). However, both methods focus on measuring the BMD of trabecular bone and are unable to obtain data related to cortical bone. In one opportunistic CT-based study of cancellous bone showed that an L1 CT-attenuation threshold of 160 HU or less was 90% sensitive and a threshold of 110HU was more than 90% specific for distinguishing osteoporosis from osteopenia and normal BMD (11). Another previous study reported that the cortical thickness of the L1 vertebra showed a sensitivity of 75%, a specificity of 90%, and an AUC (95% CI) of 0.903 (0.852–0.954) in differentiating between osteoporotic and non-osteoporotic groups (12). These studies indicate that both trabecular bone and cortical bone have high value in the diagnosis of osteoporosis. Most existing studies focus on trabecular bone or only a single cortical bone parameter (thickness) of the vertebrae, and there is a lack of multi-parameter quantitative research on vertebral cortical bone based on clinical routine CT. Although various imaging methods have been applied to the study of cortical bone, most studies so far have focused on the long bones of the extremities (13–16), and there are relatively few literatures on the study of cortical bone in vertebral bodies. In addition to the relative lack of attention paid to cortical bone, the main reason is that accurately separating and measuring vertebral cortical bone is quite challenging. To address this issue, we have developed an artificial intelligence software named the CT Spinal Bone Quantitative System (registered software number: 12626309), which enables accurate automatic segmentation and quantitative analysis of vertebral cortical and trabecular bone (12, 17). The present study aimed to explore the changing trends of cortical bone quantitative parameters of the first lumbar vertebrae (L1) measured by the CT spinal bone quantitative system with age, sex, and volume bone mineral density(vBMD), as well as their predictive value in osteoporosis.

## Methods

### Subjects and general information

A retrospective analysis was performed on 169 participants who underwent simultaneous chest-abdominal or lumbar spine CT and QCT scans at the Second Affiliated Hospital of Shandong First Medical University from July 2021 to December 2022. Inclusion criteria were as follows (10, 18): (1)Both male and female people aged ≥ 50 years, with females being in postmenopausal status; (2)Having undergone both non-contrast chest-abdomen or lumbar spine CT and QCT examinations; (3)The L1 and L2 vertebrae were completely included in the scanning range. Exclusion criteria were as follows (12, 17): (1) Presence of fractures or tumors in the thoracic or lumbar spine; (2) Suffering from bone metabolism-affecting diseases such as diabetes, thyroid diseases, etc., or taking anti-osteoporosis drugs; (3)Having undergone spinal surgery that affects the measurement of L1 and L2 vertebrae; (4)The CT image quality is unqualified (with artifacts affecting) and cannot be used for parameter analysis.

The included patients mainly underwent chest-abdominal CT for clinical evaluation of respiratory, digestive or abdominal organ diseases (such as pneumonia, liver and gallbladder diseases, abdominal space-occupying lesions), and lumbar spine CT for evaluation of low back pain, intervertebral disc herniation and other spinal diseases. In our institution, QCT can be performed simultaneously with routine CT scanning without additional radiation exposure to patients.

L1 is the upper lumbar vertebra with a relatively regular anatomical structure, free from the influence of sacroiliac joint and pelvic bone structures, and its cortical bone morphology is less affected by mechanical stress from the lower limbs. In addition, L1 is a common scanning site in clinical chest-abdominal and lumbar spine CT examinations, with high clinical accessibility and can well reflect the systemic bone metabolic status, which is widely used as a representative site for osteoporosis assessment in relevant imaging studies (5, 6).

### Quantitative parameters measurement of cortical bone of L1 vertebral body(L1_cortical bone_)

All participants underwent chest-abdomen or lumbar spine CT and QCT by using a Revolution CT scanner (GE Healthcare, USA). Scanning parameters were as follows: tube current = 400mAs, tube voltage = 120 kV, slice thickness = 1.25 mm, slice interval = 1.25 mm, matrix = 512 × 512.

**Figure 1:**
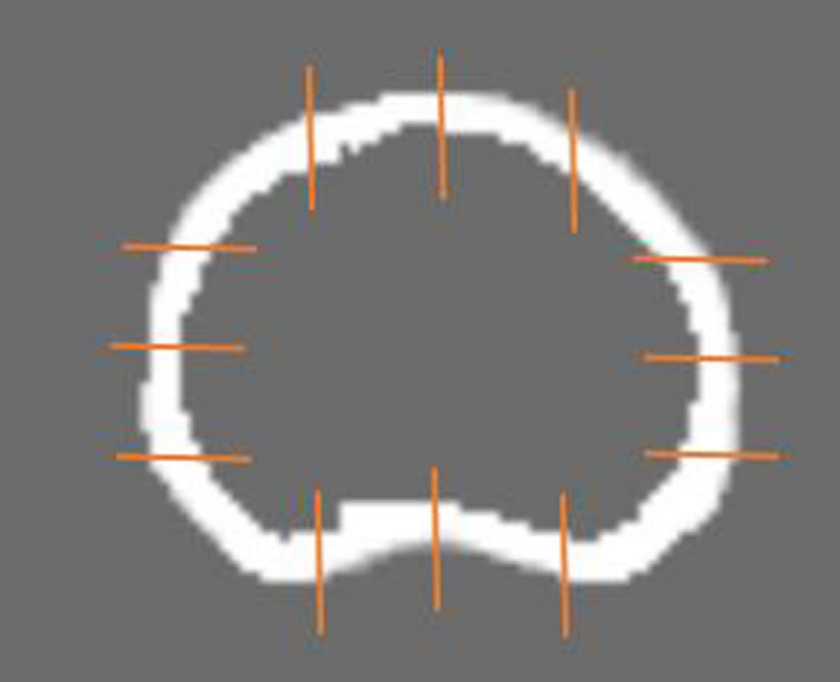
The image shows the segmentation result of the L1 cortical bone and measurement method for the cortical thickness of L1.

L1 vertebral bodies of all participants were reconstructed into slices with a thickness of 1.25 mm under bone window settings (window level = 1500HU, window width = 500HU) and then imported into the CT spine bone quantitative system for quantitative assessment (Figure 1). The average density (HU/mm^3^), average thickness (mm), average area (mm^2^) and the total volume (mm^3^) of L1_cortical bone_ were obtained using the system, respectively. The average density of cortical bone is defined as the ratio of total density to total volume of L1 vertebral body. The average thickness of cortical bone was calculated as follows: firstly, take 12 points from each slice to measure the thickness (Figure 1); secondly, calculate the average of the 12 measured value as the thickness of cortical bone of each slice; finally, take the average thickness of all slices to obtain the average thickness of L1_cortical bone_. The average area of cortical bone is defined as follows: calculate the cortical area of each layer of the L1 vertebral body, then take the average of the areas of all layers to obtain the average cortical area of the L1 vertebral body. The total volume of cortical bone is defined as the volume of L1_cortical bone_.

### Measurement of vBMD

The vBMD of L1 and L2 vertebrae was measured using QCT (Mindways, USA, Pro V5.0). The thin-slice QCT sequences were transferred to a dedicated QCT workstation for the measurement of vertebral vBMD at L1-L2. For image positioning, a crosshair was placed at the center of the vertebral body, with horizontal lines aligned parallel to the superior and inferior endplates. An elliptical region of interest (ROI) with an area of 90–110 mm^2^was then drawn to maximally include trabecular bone, while carefully avoiding osteophytes, the vertebral venous plexus, and cortical bone. Measurements were performed under the bone window (window level = 1,500 HU, window width = 500 HU). Acquisition parameters were kept consistent throughout CT scanning: tube current = 400 mAs, tube voltage = 120 kV, and slice thickness = 1.25 mm. The selected ROIs were automatically analyzed by the QCT software to derive the vBMD values. In addition, according to the expert consensus on osteoporosis diagnosis using QCT (10), the average vBMD of the L1 and L2 vertebrae was used as the final vBMD value.

The measurements were completed by two senior radiologists with more than 10 years of clinical experience in CT and QCT image analysis, both of whom have obtained the national medical imaging diagnosis qualification certificate and have received professional training in BMD measurement and CT spinal bone quantitative system operation.

### Details of grouping

1. Age grouping: All participants, male and female subgroup were grouped into three age ranges: 50–59 years, 60–69 years, and ≥70 years.
2. Osteoporosis grouping: ① The non-osteoporosis group refers to those with vBMD > 80 mg/cm^3^ (among them, the normal subgroup refers to those with vBMD > 120 mg/cm^3^; the osteopenia subgroup refers to those with 80 mg/cm^3^ ≤ vBMD ≤ 120 mg/cm^3^); ② The osteoporosis group refers to those with vBMD < 80 mg/cm^3^.

### Statistical analysis

The Med Calc 15.8 (Mariakerke, Belgium) and IBM SPSS 23.0 (Armonk, NY) software were used for statistical analysis. The descriptive statistics of all variables were expressed as mean ± standard deviation (SD). Continuous variables were tested for homoscedasticity using the Levene’s test. A one-way analysis of variance (ANOVA) was used to compare the difference among the three groups and multiple comparisons were evaluated using the Least Significant Difference (LSD) test. Receiver operating characteristic (ROC) curve analysis was performed to identify the optimal cutoff of quantitative parameters for the prediction of osteoporosis. Area under curve (AUC), specificity, and sensitivity were calculated according to the cutoff value that maximized the Youden index. DeLong test was used for AUC comparison. *p* value <0.05 was considered indicative of a statistically significant difference for all tests.

## Results

**Figure 2:**
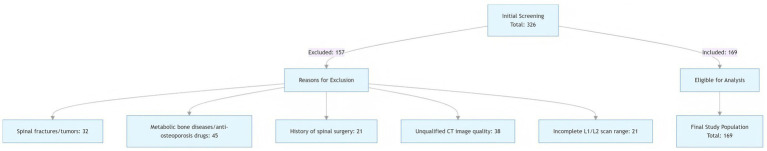
STROBE flow diagram for the study.

A total of 326 patients who underwent both chest-abdominal/lumbar spine CT and QCT examinations during the study period were initially screened; 157 patients were excluded according to the exclusion criteria (32 with spinal fractures/tumors, 45 with bone metabolic diseases/anti-osteoporosis drug use, 21 with spinal surgery history, 38 with unqualified CT image quality, 21 with incomplete scanning range of L1/L2). Finally, 169 patients were included in the statistical analysis, comprising 96 males and 73 females, with a mean age of 63.23 ± 8.41 years, and the age range was 50–87 years. Figure 2 shows the STROBE flow diagram for the study.

### Comparison of cortical bone quantitative parameters among different age groups

In the all participants group of 169 cases enrolled in this study, 71, 55 and 43 individuals were distributed in the 50–59 years, 60–69 years, and ≥70 years age groups, respectively. Among the 73 female cases, 29, 23, and 21 cases were enrolled in the above three age groups, respectively. In the all participants and the female subgroup, the average thickness, average area, and total volume of L1_cortical bone_ all showed a decreasing trend with increasing age, and the overall differences among the three age groups were statistically significant(*p* < 0.05, Tables 1, 2). However, no statistically significant difference was observed in the average density of cortical bone among the three age groups (*p* > 0.05, Tables 1, 2).

**Table 1:** Comparison of cortical bone quantitative parameters among different age groups in all participants.

Cortical bone quantitative parameters	50 ~ 59 (*N* = 71)	60 ~ 69 (*N* = 55)	≥70 (*N* = 43)	*F*	*P*
Average density (HU/mm^3^)	432.35 ± 109.70	410.11 ± 91.81	431.77 ± 96.28	0.885	0.415
Average thickness (mm)	1.83 ± 0.66	1.60 ± 0.70	1.27 ± 0.68	9.194	<0.001^bc^
Average area (mm^2^)	390.63 ± 226.90	328.60 ± 227.18	245.05 ± 169.77	6.217	0.002^c^
Total volume (mm^3^ × 10^3^)	10.46 ± 6.31	8.37 ± 6.11	6.31 ± 4.47	6.944	0.001^ac^

^a^*P* < 0.05 for differences in changes between 50–59 years versus 60–69 years.

^b^*P* < 0.05 for differences in changes between 60–69 years versus ≥70 years.

^c^*P* < 0.05 for differences in changes between 50–59 years versus ≥70 years.

ANOVA for the comparison of multiple groups of parameters, LSD test for multiple comparisons.

HU, hounsfield unit.

**Table 2:** Comparison of cortical bone quantitative parameters among different age groups in females.

Cortical bone quantitative parameters	50 ~ 59 (*N* = 29)	60 ~ 69 (*N* = 23)	≥70 (*N* = 21)	*F*	*P*
Average density (HU/mm^3^)	437.37 ± 128.00	397.26 ± 93.28	427.30 ± 83.11	0.956	0.390
average thickness (mm)	1.81 ± 0.60	1.48 ± 0.76	1.01 ± 0.62	8.965	<0.001^bc^
average area (mm^2^)	383.13 ± 239.53	254.60 ± 187.96	216.85 ± 192.25	4.383	0.016^ac^
total volume (mm^3^ × 10^3^)	10.04 ± 6.42	6.20 ± 4.64	5.39 ± 4.91	5.292	0.007^ac^

^a^*P* < 0.05 for differences in changes between 50–59 years versus 60–69 years.

^b^*P* < 0.05 for differences in changes between 60–69 years versus ≥70 years.

^c^*P* < 0.05 for differences in changes between 50–59 years versus ≥70 years.

ANOVA for the comparison of multiple groups of parameters, LSD test for multiple comparisons.

HU, hounsfield unit.

Among the 96 male cases, 42, 32, and 22 individuals were allocated to the 50–59 years, 60–69 years, and ≥70 years age groups, respectively. With increasing age, the average thickness, average area, and total volume of L1_cortical bone_ all showed a downward trend. However, no statistically significant difference were observed in the average density, average thickness, average area, and total volume of L1_cortical bone_ among the three age groups (*p* > 0.05, Table 3).

**Table 3:** Comparison of cortical bone quantitative parameters among different age groups in males.

Cortical bone quantitative parameters	50 ~ 59 (*N* = 42)	60 ~ 69 (*N* = 32)	≥70 (*N* = 22)	*F*	*P*
Average density (HU/mm^3^)	428.88 ± 96.58	419.36 ± 91.10	436.03 ± 109.18	0.198	0.820
Average thickness (mm)	1.84 ± 0.70	1.68 ± 0.65	1.51 ± 0.65	1.743	0.181
Average area (mm^2^)	395.80 ± 220.56	381.79 ± 240.51	271.97 ± 144.53	2.627	0.078
Total volume (mm^3^ × 10^3^)	10.76 ± 6.30	9.94 ± 6.62	7.18 ± 3.91	2.643	0.076

ANOVA for the comparison of multiple groups of parameters.

HU, hounsfield unit.

### Comparison of cortical bone quantitative parameters among different vBMDs

According to the diagnostic criteria of QCT for osteoporosis, among the all participants, there were 59 cases in the normal group, 58 cases in the osteopenia group, and 52 cases in the osteoporosis group. With the decrease of vBMD, the average density, average thickness, average area, and total volume of L1_cortical bone_ all showed a decreasing trend. There were statistically significant differences in the average thickness, average area, and total volume of cortical bone among the normal, osteopenia, and osteoporosis groups (*p* < 0.05, Table 4), while there was no statistically significant difference in the average density of the cortical bone among the three groups (*p* > 0.05, Table 4).

**Table 4:** Comparison of cortical bone quantitative parameters among different vBMDs.

Cortical bone quantitative parameters	Normal (*N* = 59)	Osteopenia (*N* = 58)	Osteoporosis (*N* = 52)	*F*	*P*
Average density (HU/mm^3^)	446.95 ± 114.98	414.49 ± 94.66	411.71 ± 86.70	2.200	0.114
Average thickness (mm)	1.85 ± 0.70	1.75 ± 0.61	1.19 ± 0.63	16.436	<0.001^ab^
Average area (mm^2^)	416.75 ± 217.28	365.61 ± 221.03	202.91 ± 159.19	16.507	<0.001^ab^
Total volume (mm^3^ × 10^3^)	11.11 ± 5.87	9.56 ± 6.11	5.09 ± 4.25	17.482	<0.001^ab^

^a^*P* < 0.05 for differences in changes between osteopenia versus osteoporosis.

^b^*P* < 0.05 for differences in changes between normal versus osteoporosis.

ANOVA for the comparison of multiple groups of parameters, LSD test for multiple comparisons.

HU, hounsfield unit.

### Efficacy of cortical bone quantitative parameters in distinguishing osteoporosis from non-osteoporosis

**Figure 3:**
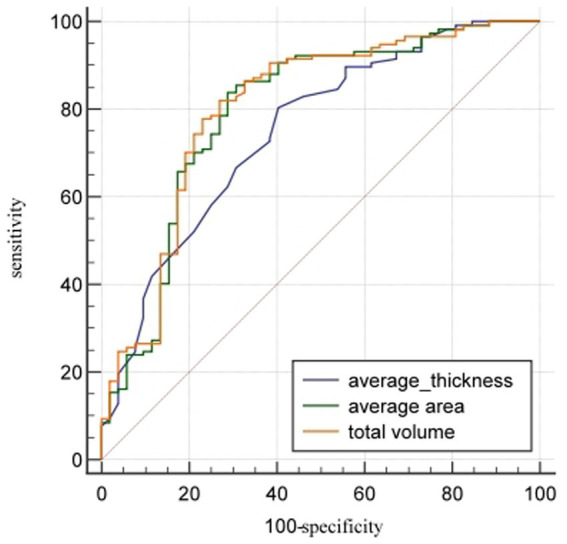
ROC comparison of cortical bone quantitative parameters in distinguishing osteoporosis from non-osteoporosis in all participants. The AUCs (95%CI) of the average thickness, average area, and total volume of cortical bone of L1 vertebral body in distinguishing osteoporosis from non-osteoporosis were 0.75 (0.68–0.81), 0.80 (0.73–0.85), and 0.80 (0.74–0.86) respectively.

Of the 169 enrolled participants, 52 were assigned to osteoporosis group and 117 to the non-osteoporosis group. The AUCs (95%CI) of the average thickness, average area, and total volume of L1_cortical bone_ in distinguishing osteoporosis from non-osteoporosis were 0.75 (0.68–0.81), 0.80 (0.73–0.85), and 0.80 (0.74–0.86) respectively, with cut-off values of 1.20 mm, 209.70mm^2^, and 5.49 × 10^3^ mm^3^. The average area and total volume cortical bone showed similar sensitivity and specificity (both greater than 70.00%) in distinguishing osteoporosis from non-osteoporosis (Table 5; Figure 3).

**Table 5:** Efficacy of cortical bone quantitative parameters in distinguishing osteoporosis from non-osteoporosis in all participants.

Cortical bone quantitative parameters	AUC (95%CI)	Criterion	Specificity (%)	Sensitivity (%)
Average thickness (mm)	0.75 (0.68 ~ 0.81)	1.20	59.62	80.34
Average area (mm^2^)	0.80 (0.73 ~ 0.85)	209.70	71.15	83.76
Total volume (mm^3^ × 10^3^)	0.80 (0.74 ~ 0.86)	5.49	73.08	82.05

AUC, area under the curve; CI, confidence interval.

**Figure 4:**
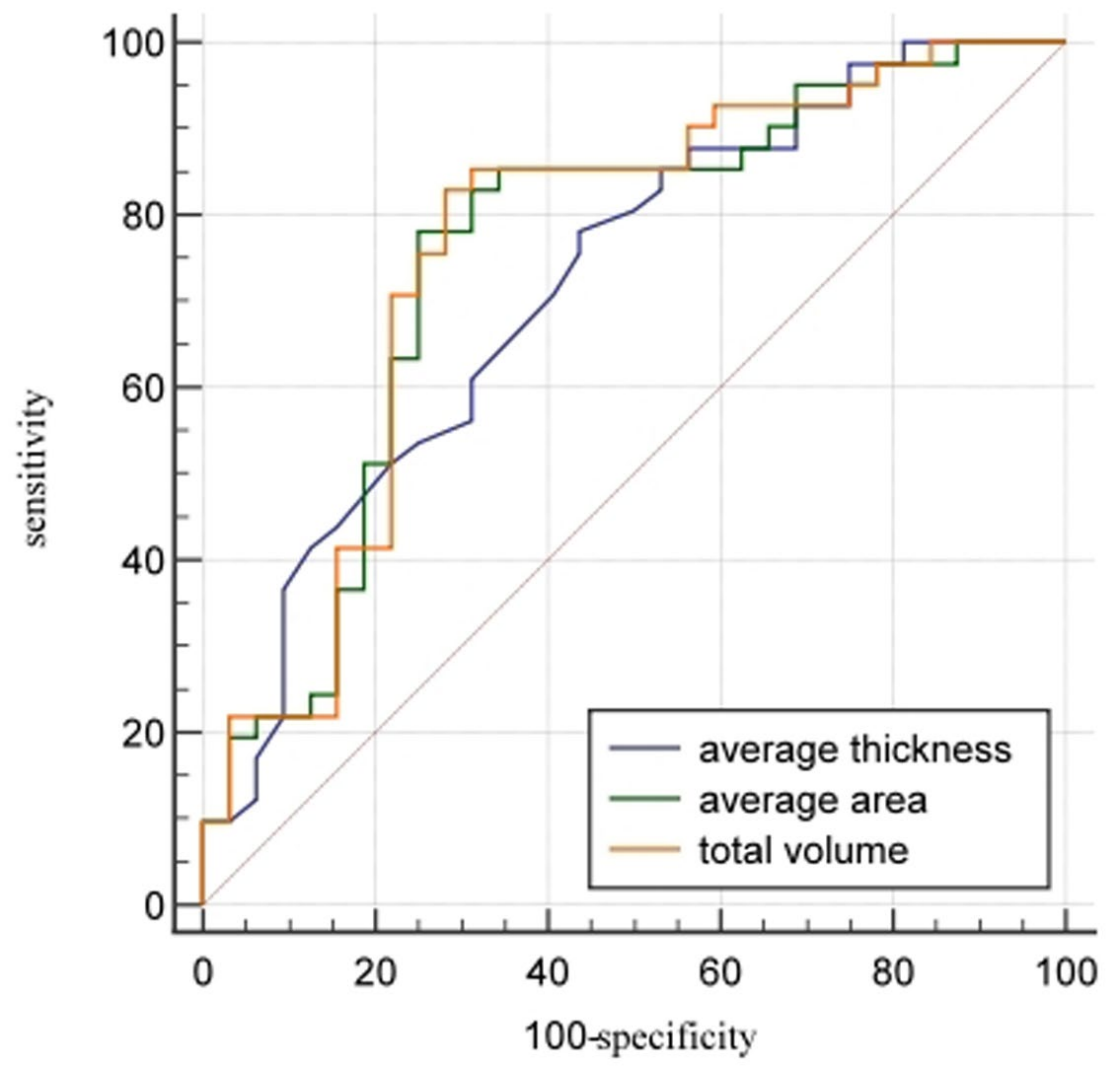
ROC comparison of cortical bone quantitative parameters in distinguishing osteoporosis from non-osteoporosis in females. The AUCs (95% CI) of the average thickness, average area, and total volume of cortical bone of L1 vertebral body in distinguishing osteoporosis from non-osteoporosis were 0.72 (0.60–0.82), 0.75 (0.64–0.85), and 0.76 (0.65–0.85) respectively.

Among the enrolled female subgroup, 32 were assigned to osteoporosis group and 41 to the non-osteoporosis group. The AUCs (95%CI) of the average thickness, average area, and total volume of L1_cortical bone_ in distinguishing osteoporosis from non-osteoporosis were 0.72 (0.60–0.82), 0.75 (0.64–0.85), and 0.76 (0.65–0.85) respectively, with cut-off values of 1.10 mm, 202.40mm^2^, and 4.46 × 10^3^ mm^3^. In terms of distinguishing between the osteoporosis and the non-osteoporosis group, the total volume of cortical bone had the highest sensitivity (82.93%), while the average area of cortical bone had the highest specificity (75.00%) (Table 6; Figure 4).

**Table 6:** Efficacy of cortical bone quantitative parameters in distinguishing osteoporosis from non-osteoporosis in females.

Cortical bone quantitative parameters	AUC (95%CI)	Criterion	Specificity (%)	Sensitivity (%)
Average thickness (mm)	0.72 (0.60 ~ 0.82)	1.10	56.25	78.05
Average area (mm^2^)	0.75 (0.64 ~ 0.85)	202.40	75.00	78.05
Total volume (mm^3^ × 10^3^)	0.76 (0.65 ~ 0.85)	4.46	71.87	82.93

AUC, area under the curve; CI, confidence interval.

**Figure 5:**
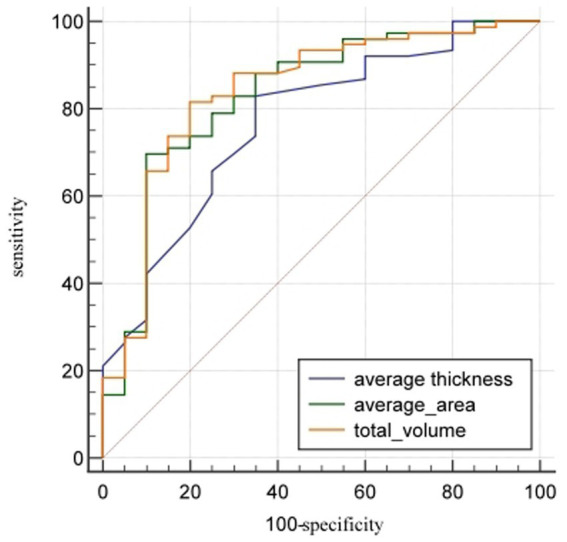
ROC comparison of cortical bone quantitative parameters in distinguishing osteoporosis from non-osteoporosis in males. The AUCs (95% CI) of the average thickness, average area, and total volume of cortical bone of L1 vertebral body in distinguishing osteoporosis from non-osteoporosis were 0.77 (0.67–0.85), 0.83 (0.74–0.90), and 0.84 (0.75–0.91), respectively.

Among the enrolled male subgroup, 20 were assigned to the osteoporosis group and 76 to the non-osteoporosis group. The AUCs(95%CI) of the average thickness, average area, and total volume of L1_cortical bone_ in distinguishing osteoporosis from non-osteoporosis were 0.77 (0.67–0.85), 0.83 (0.74–0.90), and 0.84 (0.75–0.91) respectively, with cut-off values of 1.20 mm, 283.40mm^2^, and 6.00 × 10^3^ mm^3^. In terms of distinguishing between the osteoporosis and the non-osteoporosis group, the average thickness of cortical bone had the highest sensitivity (82.89%), while the average area of cortical bone had the highest specificity (90.00%) (Table 7; Figure 5).

**Table 7:** Efficacy of cortical bone quantitative parameters in distinguishing osteoporosis from non-osteoporosis in males.

Cortical bone quantitative parameters	AUC (95%CI)	Criterion	Specificity (%)	Sensitivity (%)
Average thickness (mm)	0.77 (0.67–0.85)	1.20	65.00	82.89
Average area (mm^2^)	0.83 (0.74–0.90)	283.40	90.00	69.74
Total volume (mm^3^ × 10^3^)	0.84 (0.75–0.91)	6.00	80.00	81.58

AUC, area under the curve; CI, confidence interval.

The results of the DeLong test showed that there were no statistically significant differences in the AUCs of the three cortical bone parameters among the total participants, female subgroup, and male subgroup (*p* > 0.05, Table 8).

**Table 8:** Results of DeLong test for comparing AUCs of cortical bone quantitative parameters in osteoporosis prediction.

Study group	Comparison of cortical bone parameters	Z	*P*
All participants	Average thickness vs. average area	1.064	0.287
	Average thickness vs. total volume	1.224	0.221
	Average area vs. total volume	1.382	0.167
Females	Average thickness vs. average area	0.548	0.584
	Average thickness vs. total volume	0.652	0.515
	Average area vs. total volume	0.955	0.339
Males	Average thickness vs. average area	0.884	0.377
	Average thickness vs. total volume	1.003	0.316
	Average area vs. total volume	0.826	0.409

DeLong test for AUC comparison. AUC, area under the curve.

## Discussion

Bone strength is primarily determined by two key factors: bone mass and bone quality. Bone mass per unit volume can reflect 50–70% of the bone strength. Bone quality, on the other hand, encompasses factors such as bone metabolic turnover, bone microstructure damage, and bone mineralization, which reflects the biomechanical characteristics of bones (19). Currently, the diagnosis of osteoporosis relies on BMD-related examinations, such as DXA and QCT. Although DXA is widely recognized as a gold-standard technique for osteoporosis assessment, it measures only areal bone mineral density (aBMD), which can be influenced by patient body size and variations in vertebral morphology. In contrast, QCT provides vBMD and allows selective evaluation of cancellous bone, thereby avoiding measurement errors induced by osteosclerosis, vascular calcification, and other confounding factors (10, 20, 21). However, these methods cannot provide information on bone quality, nor can they accurately quantify the extent of bone loss, thereby limiting their utility in clinical evaluations of fracture risk and treatment efficacy (22).

Cortical bone is a densely packed, tightly connected bone tissue that accounts for 80% of total bone weight and forms the hard outer shell of bones. Furthermore, nearly 80% of human non-vertebral fractures are associated with cortical bone (23). Both aging and menopausal status can induce cortical bone loss, which primarily occurs on the endosteal surface. When cortical bone loss occurs, the periosteum can compensate for part of this loss by increasing bone size (diameter), thereby enhancing bone strength. This compensatory process occurs in both men and women, however, in women, it is a physiological response to estrogen deficiency during menopause (24). An imbalance in cortical bone remodeling can lead to increased cortical bone porosity, cortical thinning, and impairment of the cortical bone microstructure—i.e., deterioration of bone quality (25). While cortical bone plays a crucial role in determining bone strength, research on changes in cortical bone quality in the context of osteoporosis remains relatively scarce.

Ritzel et al. (26) compared cortical bone thickness using spinal vertebral autopsy specimens from 26 individuals with normal bone mass and 11 patients with osteoporosis. Their findings revealed that the cortical bone thickness across the entire spine was significantly lower in the osteoporosis group compared to the normal bone mass group. Another study focusing on cortical bone thickness in postmenopausal women with and without osteoporosis demonstrated that cortical bone thickness was significantly lower in the osteoporosis group than in the non-osteoporosis group (27). Additionally, cortical bone thickness exhibited a positive correlation with BMD, confirming that cortical bone undergoes changes in postmenopausal women. These studies collectively indicate that cortical bone thickness decreases in patients with osteoporosis. However, clinical research on cortical bone has thus far been largely confined to measuring the single parameter of thickness, lacking the inclusion of additional parameters to more comprehensively characterize cortical bone changes.

As a non-invasive three-dimensional imaging technique, micro-CT allows for the acquisition of cortical bone-related parameters, including cortical bone area, thickness, width, and porosity (28). Kim et al. (29) used micro-CT to demonstrate that alterations occur not only in trabecular bone quality but also in cortical bone microstructural parameters. In their study, the average cortical porosity in the sham-operated group was 0.13 ± 0.04, whereas the corresponding values in the ovariectomized group, glucocorticoid-administered group, and ovariectomized+glucocorticoid group were 0.75 ± 0.37, 0.60 ± 0.73, and 1.38 ± 1.12, respectively—representing 5.8-, 4.6-, and 10.6-fold increases compared to the sham-operated group. In contrast, parameters such as cortical bone area and thickness showed a decreasing trend in all intervention groups relative to the sham-operated group. Although micro-CT enables the acquisition of certain quantitative parameters related to cortical bone, its application is limited to living animals or isolated specimens, which precludes its use in routine clinical practice (30). In the present study, we demonstrated that by inputting conventional clinical CT images into our CT spinal bone quantification system, accurate differentiation between cortical and trabecular bone can be achieved, along with the extraction of cortical bone-specific quantitative parameters—including the average density (HU/mm^3^), average thickness (mm), average area (mm^2^), and total volume (mm^3^). This work presents a novel perspective and approach to more comprehensively evaluate bone strength in the context of osteoporosis, thereby enabling multi-angle, multi-parameter quantitative investigations into cortical bone changes.

The findings of this study indicated that in postmenopausal women, the three L1_cortical bone_ parameters—average thickness, average area, and total volume—exhibited a declining trend with advancing age, with statistically significant differences observed among the age groups. This suggests that as age increases, the cortical bone of postmenopausal women also undergoes structural changes, mainly characterized by thinning of average cortical thickness, reduction in average cortical area, and decrease in total cortical volume. Additionally, among postmenopausal women, statistically significant differences in average area and total volume were observed between the 50–59 and 60–69 year age groups. For average thickness, a statistically significant difference was noted between the 60–69 and ≥ 70 year age groups. Furthermore, all three parameters showed statistically significant differences between the 50–59 and ≥ 70 year age groups. In contrast, among men in the same age groups, there were no statistically significant differences in the aforementioned three parameters with increasing age. This implies that in men, age-related bone changes have little impact on cortical bone and may be primarily reflected in trabecular bone. The observed sex difference in results between men and postmenopausal women may be associated with the rapid decline in estrogen levels in women following menopause. Studies have shown that a rapid decline in estrogen levels diminishes its inhibitory effect on osteoclasts, tipping the balance toward bone resorption over bone formation and thereby contributing to the development of osteoporosis (31). Furthermore, reduced estrogen levels impair intestinal calcium absorption and enhance urinary calcium excretion, which collectively accelerate the progression of osteoporosis (32).

In the present study, all participants were categorized into three groups—normal, osteopenia, and osteoporosis—based on vBMD measured by QCT. The results showed that as vBMD decreased, all four parameters of L1_cortical bone_ (the average density, average thickness, average area, and total volume) exhibited a downward trend. Among these parameters, statistically significant differences were observed in the average thickness, average area, and total volume of cortical bone, while no significant difference was found in average density. This suggests that alongside reduced thickness, the average area and total volume of cortical bone also decrease with declining vBMD, which corroborates the characteristics of cortical bone changes in osteoporosis from a multi-parameter perspective. Furthermore, the average thickness, average area, and total volume of cortical bone all demonstrated good discriminatory ability between the osteoporosis and non-osteoporosis groups, with AUCs exceeding 0.70. Among male participants, the average thickness showed the highest sensitivity (82.89%) for predicting osteoporosis, and average area exhibited the highest specificity (90.00%). However, compared to the average thickness and area, the total volume yielded the highest AUC (95%CI) (0.84, 0.75–0.91), with a corresponding specificity of 80.00%, sensitivity of 81.58%, and a cut-off value of 6.00 × 10^3^ mm^3^. A similar pattern was observed among female participants.

In one of our previous studies, the specific algorithm of the cortical bone segmentation software employed in the present research, along with its rigorous validation, was elaborated in detail from a theoretical principle perspective (16). Subsequently, in another prior study, we utilized this software to investigate the variations in cortical bone thickness (assessed as a single parameter) across different age groups and evaluate its diagnostic utility in osteoporosis (12). In the current study, by leveraging the same segmentation system, we examine the changes in cortical bone through a multi-parameter lens. These parameters include average cortical bone density, average cortical bone area, average cortical bone thickness, and total cortical bone volume. Furthermore, we aim to identify the optimal parameter for osteoporosis diagnosis, this represents the key distinction between the present work and our earlier studies.

Notably, the present study utilizes a different dataset from our previous research. While there are certain similarities in the analytical methodologies, the approaches are not entirely identical; the current study constitutes a more in-depth exploration built upon the foundation of our prior work. Compared with our earlier investigations, the innovation of this study lies in its adoption of a multi-parameter approach to explore cortical bone changes. This methodology enables a more comprehensive reflection of the microscopic alterations in cortical bone from multiple dimensions and facilitates the identification of the most effective diagnostic parameter.

It is worth noting and considering that the potential influence of biological variability and heterogeneity within the study population may affect the measured parameters. First, Individual differences in bone metabolism (e.g., sex-related changes in osteoblast/osteoclast activity) may cause subtle cortical bone structure variations even in the same age group—for example, differing bone turnover rates between men and postmenopausal women may affect cortical bone thickness/density and further impact parameter AUCs in ROC analysis. Short-term physiological variations (e.g., hormone levels, calcium/vitamin D intake) may also introduce minor fluctuations. Second, regarding population heterogeneity, despite stratifying by sex and age, subgroup inherent differences (e.g., BMI, physical activity, smoking)—known to affect bone density—persisted. Additionally, subclinical osteoporosis, undiagnosed early bone lesions, and comorbidities (e.g., hypertension) further contributed to heterogeneity by altering bone metabolism and morphology.

The strengths of the study need to be emphasized: (1) The study uses a self-developed CT spinal bone quantitative system to realize the accurate automatic segmentation and multi-parameter quantitative analysis of vertebral cortical bone, which solves the technical difficulty of vertebral cortical bone measurement; (2) The study analyzes the differences in cortical bone parameters between men and women and different age groups, and clarifies the age and sex characteristics of vertebral cortical bone changes in osteoporosis.

It is important to acknowledge the limitations of our study: (1) Methodological limitations: this is a single-center retrospective study, which may have selection bias; the lack of long-term follow-up data cannot evaluate the predictive value of cortical bone parameters for osteoporotic fractures; the present study did not perform a comparative analysis between quantitative parameters of cortical bone and trabecular bone in the L1 vertebral body; (2) Potential biases: the included patients are from a single hospital, and the clinical characteristics may be relatively concentrated, which may affect the representativeness of the results; (3) Generalizability concerns: the study only included Chinese patients aged ≥50 years, and the results may not be applicable to young people, children or other ethnic groups. In future research, these limitations will be taken into account and improved upon to obtain more rigorous and convincing research results. The specific details are as follows: (1) Conduct a multi-center, prospective cohort study with a larger sample size to verify the results of this study and reduce selection bias; (2) conduct long-term follow-up to explore the predictive value of cortical bone parameters for osteoporotic fractures and perform a comparative analysis between quantitative parameters of cortical bone and trabecular bone in the L1 vertebral body; (3) Expand the study population to include young people with different years and other ethnic groups to improve the generalizability of the results.

## Conclusion

The quantitative parameters including the average thickness, average area, and total volume of L1_cortical bone_ measured by the CT spinal bone quantitative system appear to show a downward trend with increasing age and decreasing vBMD. The changes of cortical bone parameters in postmenopausal women tend to be more significant than those in men with increasing age. All three quantitative parameters may potentially help distinguish patients with osteoporosis, among which the total volume of cortical bone seems to exhibit the highest predictive efficacy.

## Data Availability

The raw data supporting the conclusions of this article will be made available by the authors, without undue reservation.
